# Differentiating CSF flow artifacts from pathology: an educational review

**DOI:** 10.1186/s13244-025-02153-9

**Published:** 2025-12-22

**Authors:** Vivek Pai, Alexandre Boutet, Mikail Malik, Yash Patel, Sriranga Kashyap, Jurgen Germann, Kanchan Gupta, Bhujang Pai, Birgit Betina Ertl-Wagner, Bela Purohit

**Affiliations:** 1 Division of Neuroradiology, Department of Diagnostic and Interventional Radiology, The Hospital for Sick Children, Toronto, ON Canada; 2 Department of Medical Imaging, University of Toronto, Toronto, ON Canada; 3 Joint Department of Medical Imaging, Toronto, Ontario Canada; 4 Temerty Faculty of Medicine, University of Toronto, Toronto, ON Canada; 5 Krembil Brain Institute, University Health Network, Toronto, ON Canada; 6 Center for Advancing Neurotechnological Innovation to Application (CRANIA), Toronto, ON Canada; 7 Department of Radiology, Lilavati Hospital and Research Centre, Mumbai, India; 8 Department of Radiology, Sir H. N. Reliance Foundation Hospital and Research Centre, Mumbai, India; 9 Department of Neuroradiology, National Neuroscience Institute (NNI), Singapore, Singapore

**Keywords:** Magnetic resonance imaging, Artifacts, Cerebrospinal fluid, Neuroimaging

## Abstract

**Abstract:**

Magnetic resonance imaging (MRI) of the neuroaxis is prone to a variety of artifacts. Familiarity with these artifacts and their respective mitigation techniques is essential for accurate neuroradiological interpretation. In this educational review, we focus on artifacts caused by the physiological flow of cerebrospinal fluid (CSF), which are encountered commonly and, depending on the context, may be beneficial or detrimental in diagnostic decision-making. The pictorial examples provided will illustrate key cases with their practical implications.

**Critical relevance statement:**

This paper highlights common CSF flow artifacts, including phase encoding artifacts, time-of-flight signal loss, entry slice phenomenon, and intravoxel dephasing, emphasizing their impact on diagnosis interpretation and mitigation strategies.

**Key Points:**

- CSF artifacts stem from flow dynamics, phase differences, or magnetic field interactions.
- Artifacts obscure or mimic pathologies, degrade image quality, or occasionally aid in diagnostic decision-making.
- Mitigation strategies are simple and intuitive, including modification of phase directions, employing alternate imaging sequences, and altering MRI parameters.

**Graphical Abstract:**

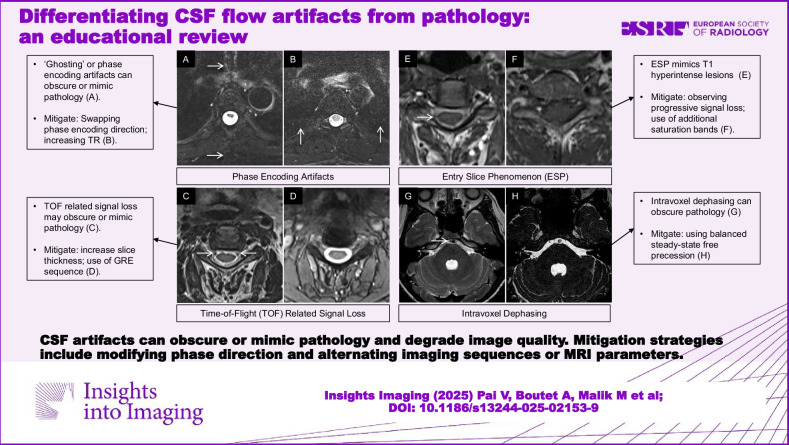

## Introduction

MRI plays a pivotal role in neuroimaging, but is also subject to artifacts. Patient movement and susceptibility from metallic hardware are frequently encountered artifacts in clinical practice and have been extensively discussed in radiological literature [1, 2]. However, in the context of neuroradiology, cerebrospinal fluid (CSF) flow artifacts are ubiquitous, and may obscure anatomic visualization or hinder diagnostic decision-making. Nevertheless, CSF flow can be useful in neuroadiological interpretation in certain situations, with an appropriate understanding of CSF flow physiology and the physics of artifact genesis. In this educational review, we briefly discuss CSF flow dynamics and provide an illustrative review of the common artifacts related to CSF flow. Simple and intuitive methods of rectification, with examples of the potential pitfalls and some clinical utility of these artifacts, are also provided.

## CSF flow physiology

CSF is the clear ultrafiltrate of plasma contained within the intracranial ventricular system; it circulates in the subarachnoid spaces around the brain and spinal cord [3]. CSF plays a key role in maintaining intracranial homeostasis, transporting substances (nutrients, toxins) and providing hydromechanical support to the neuroaxis [4, 5]. It is primarily, but not exclusively, produced by the choroid plexus and tela choroidea of the ventricles at an approximate daily average of 400–600 mL in adults [4]. The majority of the CSF volume is formed in the lateral ventricles from where it flows through the foramina of Monro into the third ventricle. CSF subsequently flows through the cerebral aqueduct into the fourth ventricle. Eventually, it exits into the subarachnoid space across the foramina of Luschka and Magendie and enters the central ependymal canal through the obex [3, 4, 6]. Circulating subarachnoid CSF is reabsorbed by arachnoid granulations according to the classical model of CSF flow dynamics, though this has been challenged with a better understanding of glymphatics; proposed sites of absorption include the brain parenchyma and the peripheral lymphatics by efflux across the olfactory bulb and the cranial/spinal nerves [4, 7]. The circulation of CSF is facilitated by transmitted pulsations of the intracranial vessels and respiratory motion [4, 6, 8]. CSF net flow is mostly rostrocaudal within the ventricular system and multidirectional in the subarachnoid space [4, 9]. These directional and pulsatile flow dynamics form the fundamental basis of CSF-related artifacts on MR images [6, 10].

### Phase encoding artifacts

**Fig. 1:**
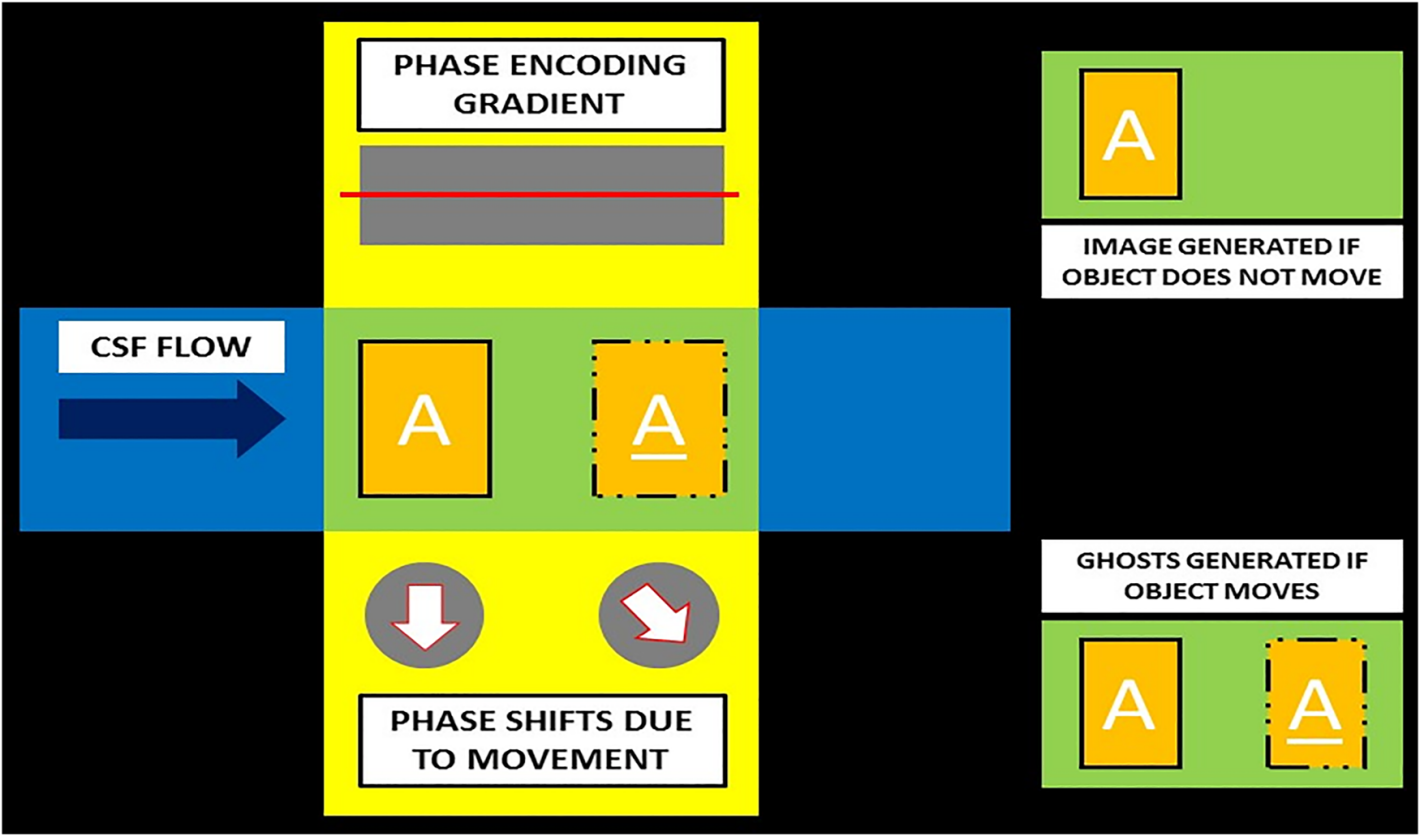
Diagrammatic representation of phase encoding artifacts. A change in the position of a spin during the application of a phase encoding gradient induces a phase difference in k-space, leading to spatial mismapping

Raw MR imaging data (k-space data) are acquired using multiple frequency-encoded samples for each phase encoding step to form a data matrix. This matrix is then converted into an image using a 2D Fourier transform [11, 12]. Movement of mobile CSF spins affects their precessional phase, causing them to acquire a new resonance frequency. This phenomenon introduces a phase difference in the k-space, thus causing spatial mismapping of the source of the signal. The net outcome of such mismapping is the formation of aliases (“*ghosts*”) of the CSF across the image, often beyond anatomic limits [2] (Fig. 1).

Phase encoding artifacts are more pronounced along the direction of the phase, rather than the frequency, encoding gradient. This is due to the prolonged data acquisition time for the former (seconds vs microseconds for the latter) [11, 13, 14]. The orientation of the artifacts aligns with the direction of the phase encoding gradient, most often along the short axis of the image. The distance separating the aliasing artifact (measured in voxels) is proportional to the image size, the number of signals averaged, and the repetition time (TR) but inversely proportional to the period of pulsation [13]. CSF ghosts are also more conspicuous when the background signal from stationary tissues is relatively lower (e.g., on fat-saturated T2-weighted sequences) [6].

**Fig. 2:**
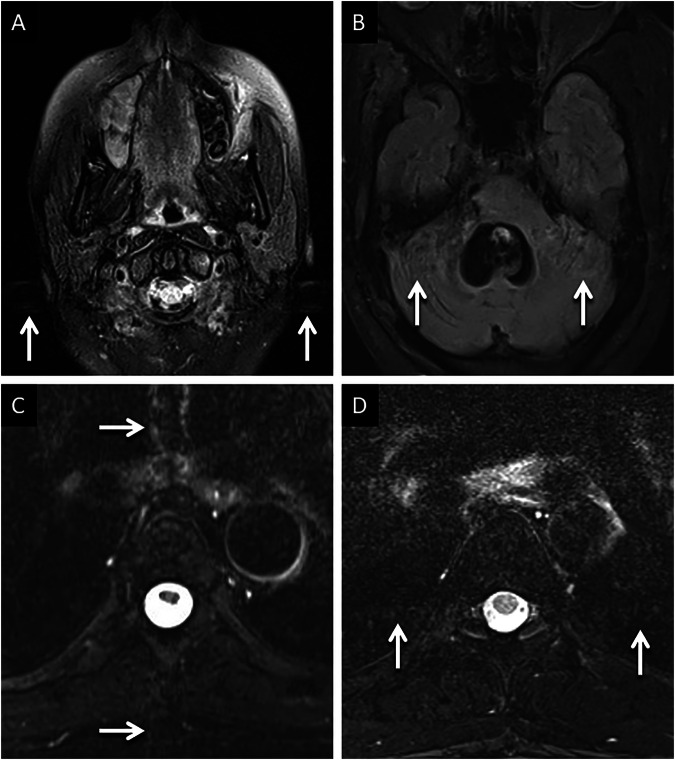
Examples of phase encoding artifacts and rectification. Typical appearances of phase encoding artifacts (**A**, **B**) in two different patients. Axial FLAIR image (**A**) in a 13-year-old presenting with headache, obtained at the level of the foramen magnum, demonstrates a linear band of relatively high signal (arrows) extending across anatomic boundaries in keeping with phase encoding artifact caused by CSF around the cervicomedullary junction. Axial FLAIR image (**B**) through the posterior fossa in a 5-year-old patient reveals ex vacuo dilatation of the fourth ventricle, due to remote surgical excision of a medulloblastoma. Note the band-like phase encoding artifact (arrows) extending across anatomic boundaries, consequent to turbulent CSF flow. Rectification of phase encoding artifacts (**C**, **D**). Axial fat-saturated T2 image (**C**) through the mid-thoracic spine, in a 35-year-old patient presenting with backache, reveals vertically oriented phase encoding artifacts (arrows) caused by pulsatile CSF flow within the thoracic thecal sac. The artifact obscures the vertebral body, limiting diagnostic interpretation. Axial fat-saturated T2 image (**D**) obtained through the same region, after swapping the direction of the phase encoding gradient and increasing TR, changes the orientation and suppresses the signal intensity of the artifact (arrows), thereby improving visualization of the adjacent vertebra

Phase-encoding artifacts can be a nuisance for radiologists as they can obscure pathologies or mimic lesions, potentially leading to missed findings or a misdiagnosis. One of the easiest rectification methods, and to improve the visualization of obscured anatomy, is swapping the directions of the phase and frequency encoding [6]. This realigns the in an orthogonal plane [6]. Increasing the repetition time (TR) duration can also reduce the intensity of phase encoding artifacts, though this increases scan time [6, 13]. An example of phase encoding artifacts and their rectification is provided in Fig. 2.

### Time-of-flight (TOF) related signal loss

**Fig. 3:**
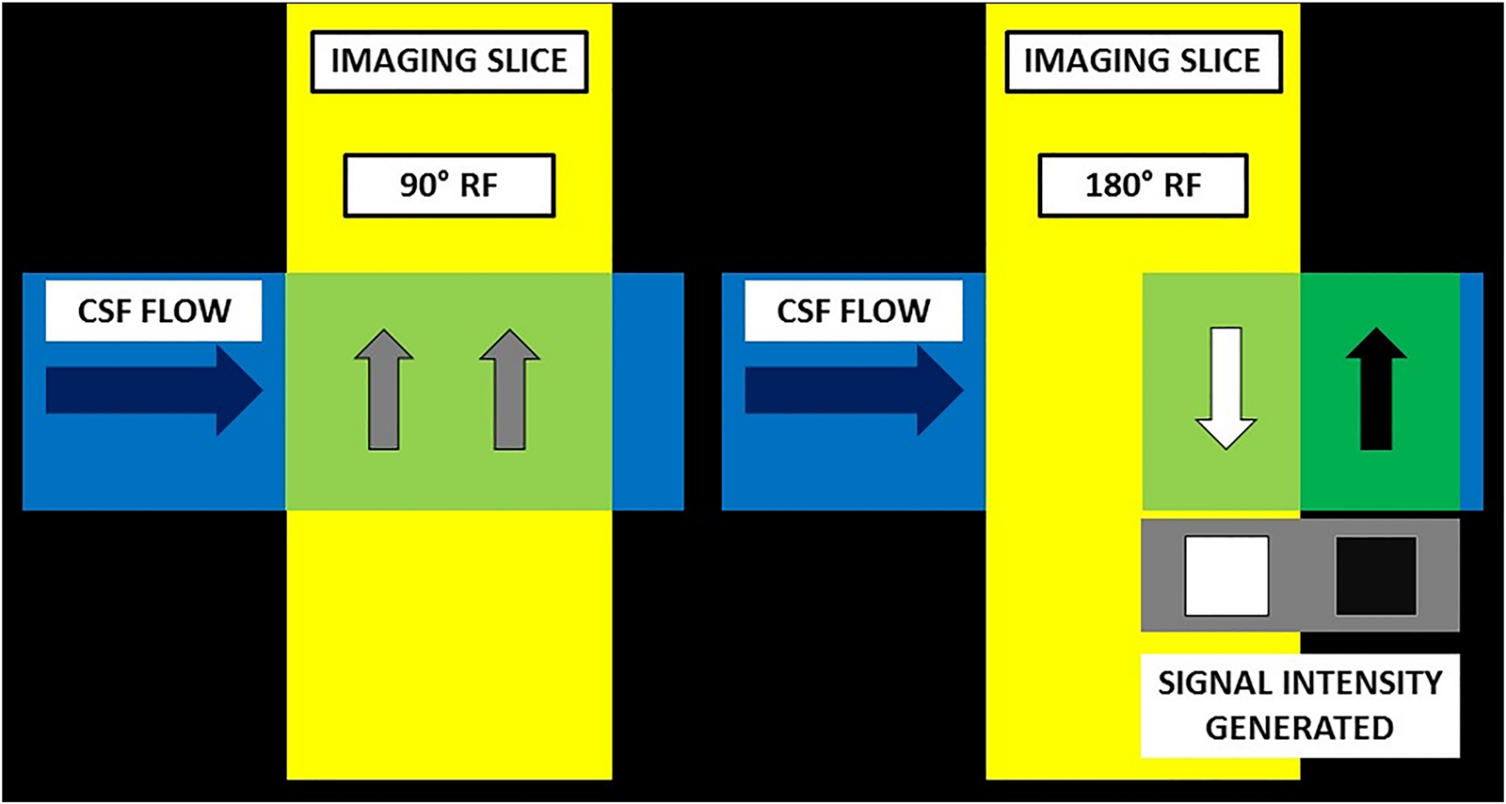
Diagrammatic representation of TOF-related signal loss. In order to generate a signal, protons must receive both 90° and 180° pulses. Mobile protons, which move out of the imaging slice between the 2 pulses, are unable to generate a signal

In spin echo sequences, protons in each slice must receive the excitation pulse (90°) and a subsequent rephasing pulse (180°) to generate a signal. Thus, stationary protons receive both pulses and generate a signal. However, mobile CSF protons may receive only the 90° pulse and may exit the imaging slice, therefore not experiencing the 180° pulse. These selectively ‘excited’ protons do not impart a signal and appear hypointense (i.e., demonstrate signal loss). This phenomenon is known as TOF-related signal loss [6, 15] (Fig. 3).

**Fig. 4:**
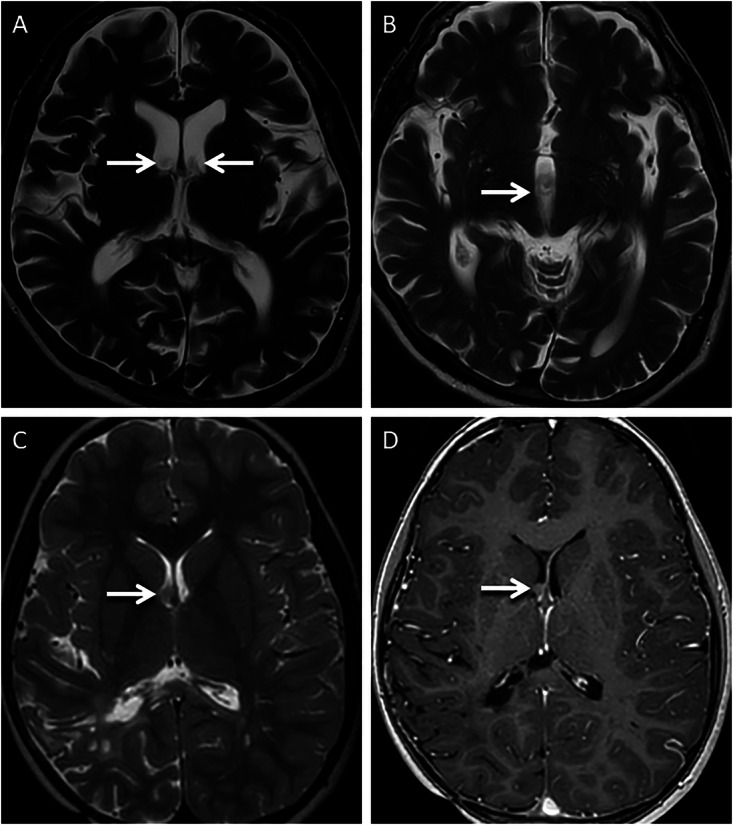
Examples of TOF-related signal loss and potential pitfalls. Typical appearances of TOF-related signal loss (A, B). Axial T2W images through the foramina of Monro (**A**) and third ventricle (**B**), in a 65-year-old presenting with loss of consciousness, reveal areas of signal loss (arrows in **A**, **B**) occurring as a result of TOF-related signal loss. Ependymal metastasis mimicking TOF-related signal loss (**C**, **D**). MR surveillance of a 6-year-old patient with previously resected supratentorial ependymoma. Axial T2 image (**C**) reveals post-treatment related changes in the right parietal lobe with a small focus of intermediate to hypointense signal (arrow) in the right foramen Monroe, which could be misinterpreted as TOF-related signal loss. Axial contrast-enhanced T1 image (**D**) obtained at the same location confirms corresponding homogeneous enhancement, thus confirming a metastatic deposit

The degree of TOF signal loss depends on the velocity of CSF flow. With increasing velocity, the proportion of the protons receiving both pulses is smaller, leading to an increase in the TOF-related signal loss [15]. This effect is amplified in certain regions of the ventricular system (e.g., foramina of Monro, fourth ventricular outlets). In addition to CSF velocity, TOF signal loss also depends on the time to echo (TE). As the TE increases, the time between the application of the 90° and 180° pulses increases, decreasing the chance of delivering both pulses to the moving protons [6]. TOF-related signal loss is also dependent on the thickness of the slice; a thicker slice increases the chance of protons experiencing both pulses and reduces the TOF signal loss [6]. Similar to phase-encoding artifacts, TOF signal loss is generally a drawback for radiological interpretation as it degrades image quality. Depending on its shape and location, these artifacts may mimic or obscure lesions. An example of TOF signal loss and its potential pitfalls are provided in Fig. 4.

**Fig. 5:**
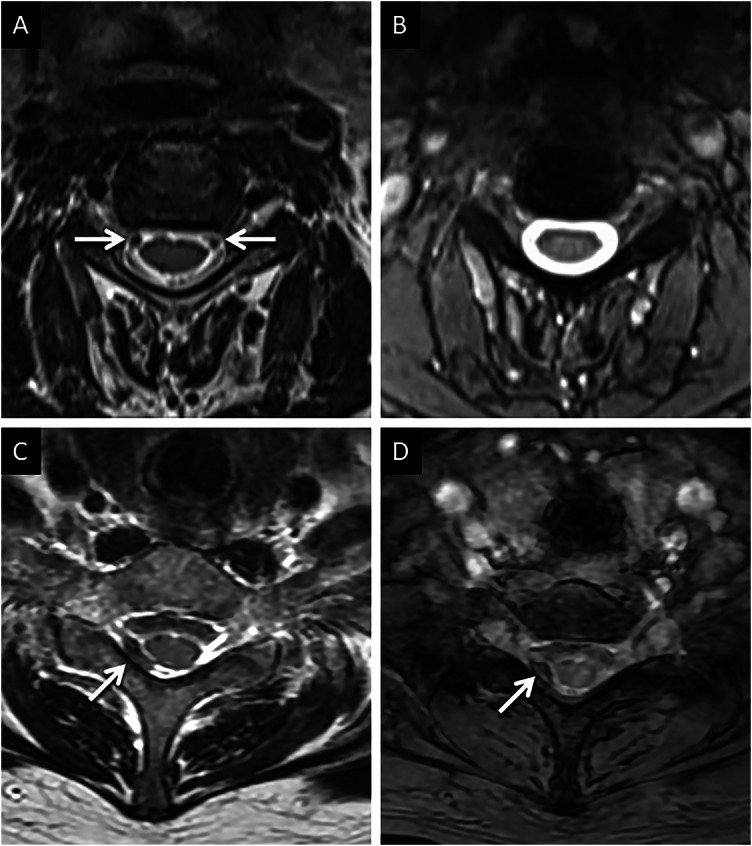
Value of GRE. Typical appearances of TOF-related signal loss and its rectification using GRE (**A**, **B**). Axial T2W image (**A**), through the cervical spine in a 30-year-old presenting with symptoms of radiculopathy, shows TOF-related signal loss (arrows), most marked along the ventrolateral aspects of the cord. Corresponding axial GRE image (**B**) shows complete rectification of the artifactual hypointensity. Appearances of hemorrhage within the cervical spine (**C**, **D**). Axial T2W image (**C**) through the cervical spine, in a 50-year-old involved in a motor vehicle collision, reveals eccentric T2 hypointensity (arrow) along the right lateral aspect of the cervical thecal sac. Axial GRE image (**D**) confirms corresponding susceptibility (arrow) in keeping with hemorrhagic products

Supplementing spin-echo sequences with gradient echo sequences (GRE) is a common method to eliminate TOF-related signal loss. In GRE sequences, the refocusing is performed using a non-slice-selective gradient instead of the slice-selective refocusing (180°) pulse used in spin echo sequences [15, 16]. In other words, the excited protons are refocused irrespective of their location in the imaging volume [15]. Additionally, the short repetition time (TR) saturates protons in stationary structures, increasing the contrast with the CSF [15]. GRE, also by virtue of their inherent sensitivity to field inhomogeneities, help differentiate TOF-related artifacts from paramagnetic lesions (calcification, hemorrhage), both of which appear hypointense on T2-weighted images [17]. The value of GRE in distinguishing TOF-related signal loss from a true pathology is exemplified in Fig. 5.

### Entry slice phenomenon (ESP)

**Fig. 6:**
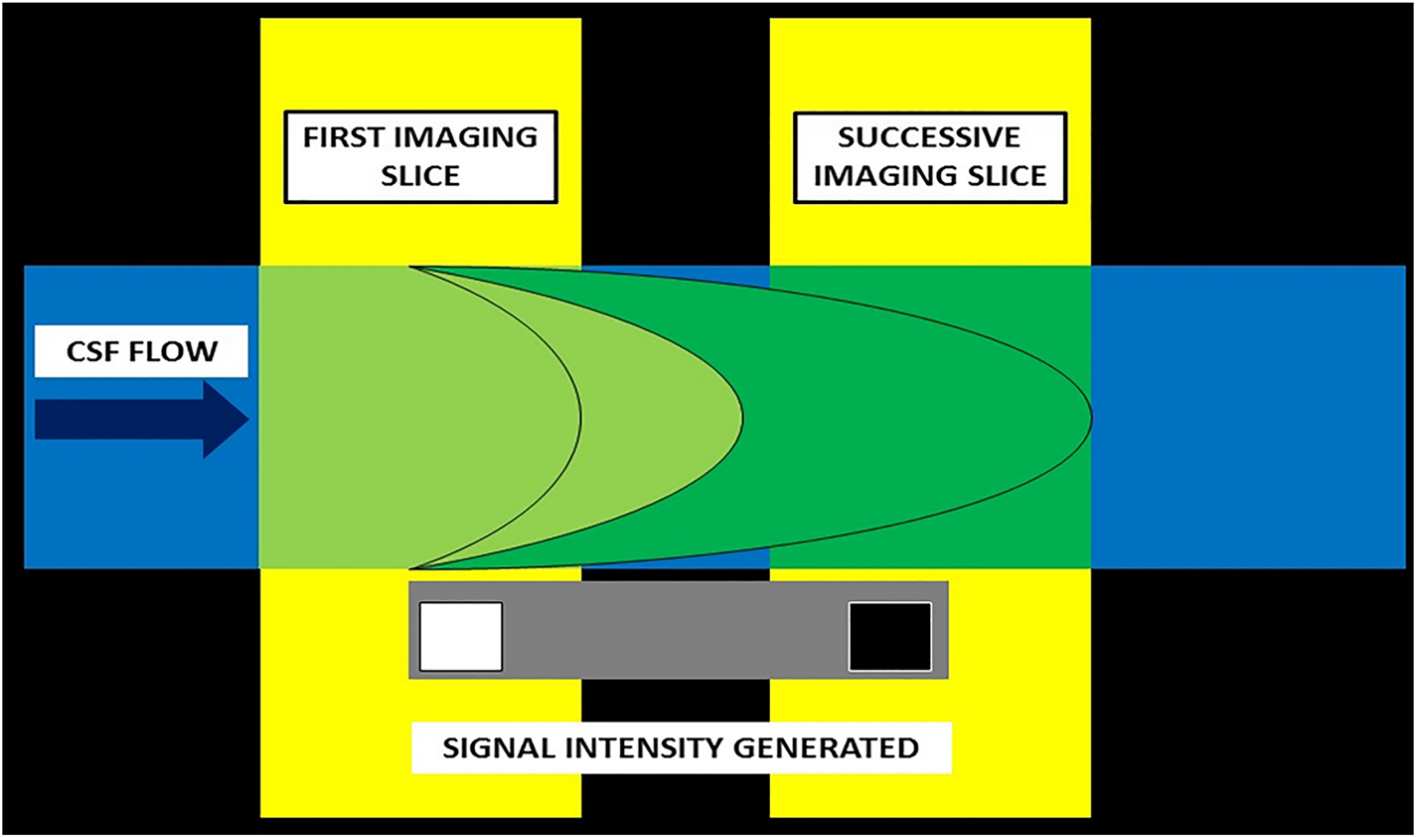
Diagrammatic representation of ESP. In pulse sequences employing a short TR, stationary spins are subject to multiple short interval RF pulses, leading to signal saturation. However, when unsaturated ‘fresh’ spins flow into the imaging plane, they experience the TR signal for the first time and are flipped into the transverse plane, therefore emitting signals. Progressively, the mobile spins get saturated as well

During the acquisition of sequences employing a short TR (i.e., short interval 90° pulse), the time for longitudinal recovery is not long enough, and the magnetic moments tend to align parallel to the magnetic field (i.e., spin down). Hence, stationary protons are saturated. On the contrary, fully relaxed protons of inflowing CSF are ‘fresh’ with the magnetic moments aligned in the spin-up direction, imparting a bright signal in the initial imaging slices as they receive the radiofrequency pulse. With successive slices, the ‘fresh’ protons become saturated due to the effects of the short TR, and the bright signal fades away to be replaced by iso- and even hypointense signals. This phenomenon of differential signal intensity, imparted by mobile CSF, is known as the ESP (also called in-flow artifact) [15, 18, 19] (Fig. 6). The shorter the TR, the faster the saturation of the flowing protons and the lesser the magnitude of ESP [15]. Slice thickness also has a similar effect on the ESP; with thicker slices, more CSF experiences the excitation pulse, reducing the ESP [15].

**Fig. 7:**
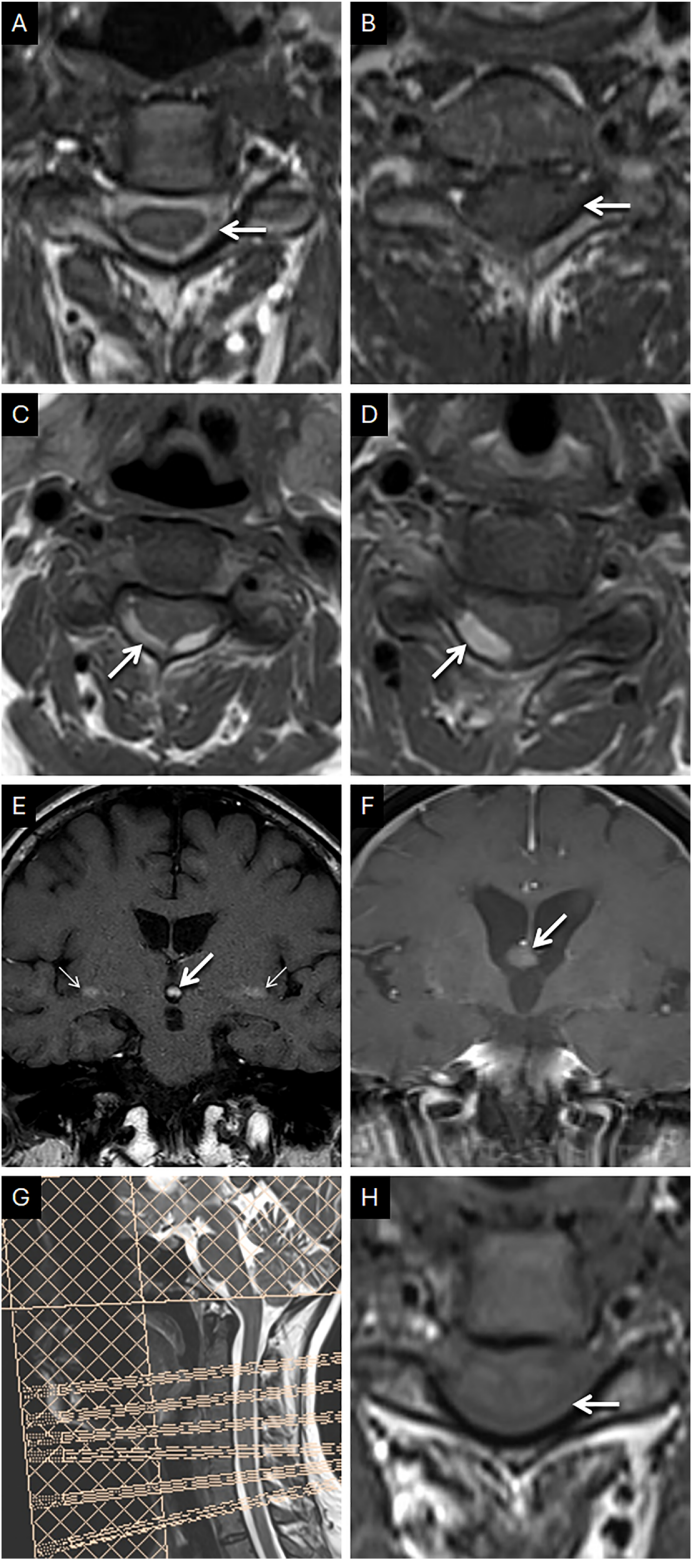
Usual appearances of ESP and its potential pitfalls along with the method of rectification. Typical appearances of ESP (**A**, **B**). Axial sequential craniocaudal T1 images (**A**, **B**) through the cervical spine, in a 35-year-old presenting with cervicogenic headache, reveal hyperintense signals within the thecal sac in the upper cervical spine (arrow in **A**) with progressive signal loss caudally (arrow in **B**). Extra-axial hemorrhage mimicking ESP (**C**, **D**). Axial sequential craniocaudal T1 images (**C**, **D**) through the cervical spine, in a 20-year-old presenting with polytrauma, reveal hyperintense signals (arrows) along the cervical cord representing subdural hemorrhage. Note the lack of progressive fading of the intensity (compared to flowing CSF in **A**, **B**). Pitfalls in interpreting ESP (**E**, **F**). Coronal T1 image (**E**) in a 16-year-old presenting with precocious puberty reveals a focus of hyperintensity (arrow) within the third ventricle related to ESP. This artifactual signal faded out on deeper coronal slices (not shown). Note the phase encoding artifacts (thin arrows) seen in the adjacent brain parenchyma, in linear relation with the artifactual bright signal within the third ventricle, confirming CSF flow. Coronal contrast-enhanced T1 image (**F**) in a 50-year-old patient presenting with headache reveals an incidental, round, relatively hyperintense mass lesion along the roof of the third ventricle in keeping with a colloid cyst (arrow). It is important to consider the site of the lesion and the absence of adjacent phase encoding artifacts (which are only associated with mobile spins). Method of rectification of ESP (**G**, **H**) in a 40-year-old presenting with bilateral upper limb radiculopathy. Sagittal scout image (**G**) obtained for planning of a cervical spine MRI with additional saturation band across the posterior fossa to null ESP from the craniocaudal flow of CSF. Axial T1 image (**H**) acquired subsequently completely saturates CSF signals in the thecal sac (arrow)

ESP is common on T1-weighted images (short TR) [6]. This artifact can mimic intrinsically T1 hyperintense pathologies within the CSF, such as hemorrhage, particularly in the context of trauma or in the presence of an underlying lesion predisposed to hemorrhage (e.g., vascular shunting lesions) [20, 21]. The gradual fading of a hyperintense signal on subsequently obtained slices and the lack of ‘blooming’ artifact on GRE are key pointers of ESP and differentiate it from blood products. Also, saturation bands can reduce this phenomenon. They deliver 90° pulses to a volume of anatomy outside the field of view. Hence, flowing CSF protons receive an excitation pulse before and after entering the imaging stack, leading to saturation and effectively nulling the signal from flowing protons [15, 19]. Given that ESP occurs as a result of CSF flow, it may be associated with phase encoding artifacts in the vicinity, another diagnostic clue to consider the artifactual nature of hyperintensity within the CSF system. The typical appearances of ESP, its potential pitfall, and the method of rectification are demonstrated in Fig. 7.

Artifactual hyperintense signals within the ventricular system may also be encountered on FLAIR imaging. This is commonly seen in the third and fourth ventricles. It is thought to be due to the reflux of CSF and/or increased CSF inflow velocity from the lateral ventricles during the inversion delay. As a result of the high velocity in these regions, CSF protons may not experience the inversion pulse, hence remain un-nulled, and thereby impart a signal [22].

### Intravoxel dephasing

**Fig. 8:**
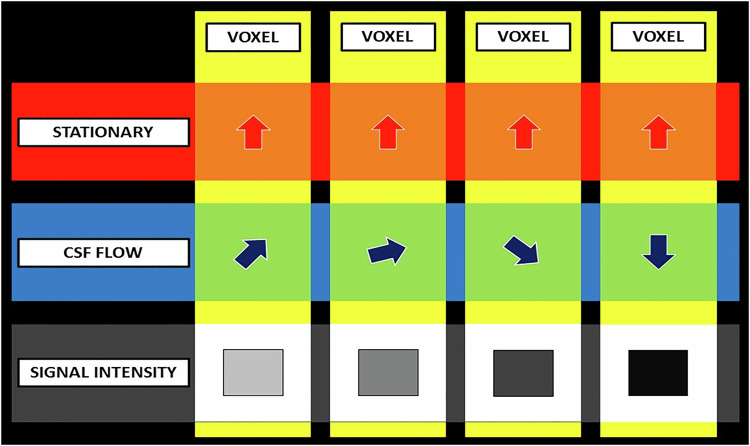
Diagrammatic representation of Intravoxel dephasing. Following the application of gradients, mobile protons may gain or lose phase. Within a voxel, coexistent stationary and flowing spins induce phase differences which results in a reduction of the signal intensity. The more the phase difference within a voxel, the more the signal loss

Mobile spins accumulate phase as they flow relative to the magnetic gradient. This phase accumulation is directly dependent on the spins’ velocity and acceleration. When stationary and mobile protons exist in a single voxel, the difference in phase results in a reduction of the signal amplitude. The resultant hypointense signal generated is known as intravoxel dephasing [6, 15, 23] (Fig. 8).

**Fig. 9:**
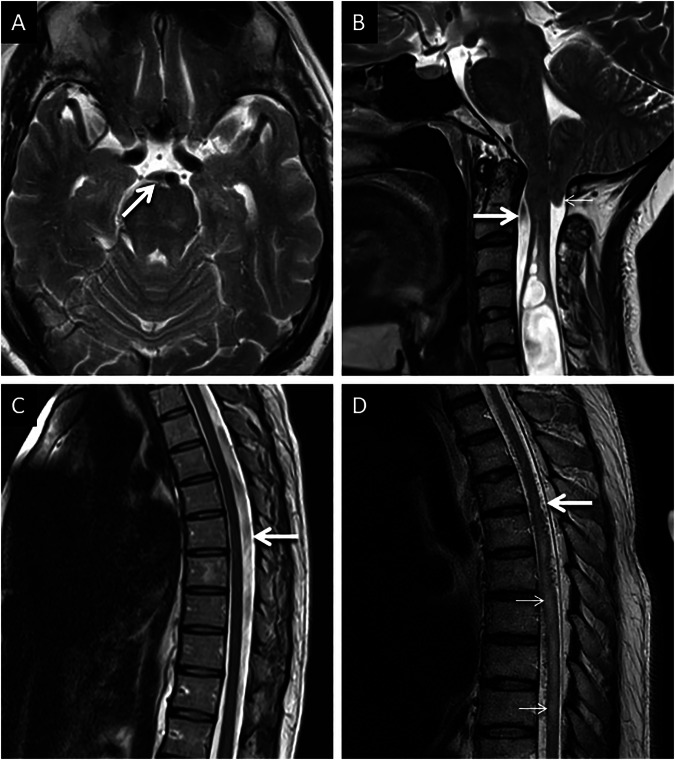
Intravoxel dephasing and potential pitfalls. Typical appearances of intravoxel dephasing (**A**–**C**). Axial T2W image (**A**), in a 75-year-old with dementia, reveals a curvilinear area of hypointensity (arrow) along the right lateral margin of the basilar trunk. This is a common location of dephasing (due to local turbulence caused by basilar pulsations). Midline sagittal T2W image (**B**), in a 9-year-old with Chiari 1 deformity, reveals crowding of the neural structures at the foramen magnum due to cerebellar tonsillar descent (thin arrow). There is narrowing of the CSF columns around the cervicomedullary junction with a jet of CSF flow seen as an area of intravoxel dephasing, within the ventral CSF column (arrow). Sagittal T2 image (**C**), in a 57-year-old with backache, reveals areas of flow-related signal loss (arrow) in the dorsal CSF column of the thoracic thecal sac. True intrathecal vascular flow voids mimicking intravoxel dephasing (**D**). Sagittal T2W image (**D**), in a 45-year-old with myelopathy, reveals multiple hypointensities along the thoracic cord (arrow). These mimic signal loss due to intravoxel dephasing, but are curvilinear and contiguous. Also note the abnormal T2 hyperintense signal within the thoracic cord (thin arrow), due to venous congestion. Digital subtraction angiogram (not shown) confirmed a dural arteriovenous fistula

A common location of intracranial intravoxel turbulence is in the prepontine cistern caused by transmitted basilar pulsations, often contained laterally by anterior pontine membranes [6, 24]. Radiologists may commonly encounter flow turbulence at the foramen magnum, especially in patients with Chiari I deformity. The tonsillar ectopia in Chiari I deformity causes complex flow patterns, resulting in eddy currents and vortices, with an increase in the peak systolic and diastolic flow velocity. CSF velocity at the foramen magnum with this condition reaches 12 cm/s compared to 5 cm/s seen in healthy adult volunteers [25]. Turbulent dephasing artifact is also common in the dorsal subarachnoid space on sagittal T2-weighted images of the thoracic spine [6]. CSF flow in this location is complex due to the combination of transmitted cardio-respiratory pulsations, cranio-caudally directed flow, and, to a lesser extent, the ventral to dorsal direction of the CSF flow [6]. The hypointense signals can simulate vascular flow voids associated with pathologies such as dural arteriovenous fistula or a mass lesion [16]. Turbulent CSF artifacts are bulky and discontinuous as opposed to the serpentine and continuous vascular flow voids [16]. Examples of common locations of intravoxel dephasing are provided in Fig. 9.

**Fig. 10:**
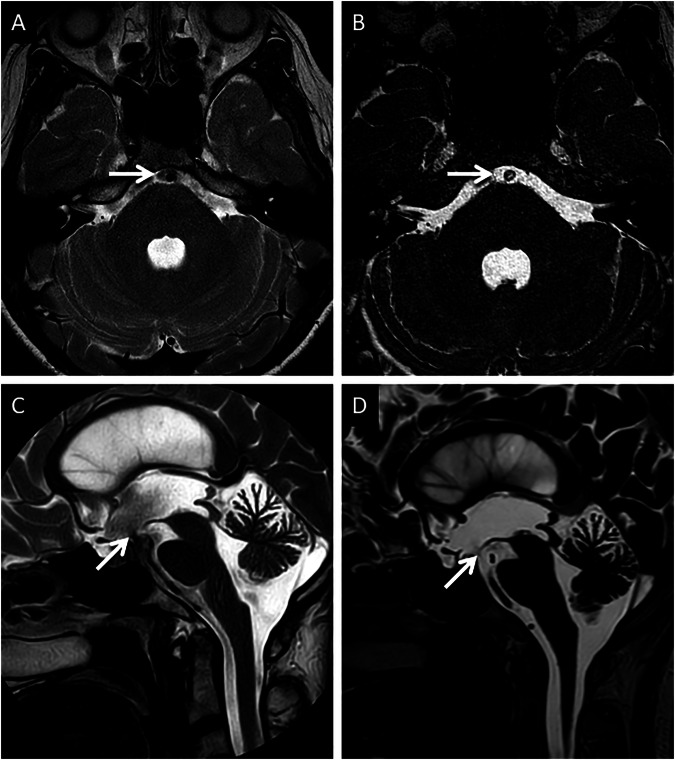
Value of FIESTA imaging in the rectification of intravoxel dephasing. Healthy volunteer / Patient 1 (**A**, **B**). Axial T2W image (**A**) reveals signal loss along the basilar trunk due to intravoxel dephasing. Axial FIESTA (**B**) rectifies the flow artifact around the basilar trunk (arrow) and improves visualization of the basal cisterns. Patient 2 (**C**, **D**), a 22-year-old imaged following ETV for triventricular hydrocephalus secondary to an aqueductal web. Sagittal T2W image (**C**) reveals intravoxel dephasing along the tuber cinereum owing to the craniocaudal flow of CSF across the surgically created defect (ETV), thereby confirming patency (arrow). Sagittal FIESTA image (**B**) demonstrates near complete rectification of the CSF dephasing and reveals the ETV defect (arrow)

Several mitigation techniques can be used. The artifact can be rectified using smaller voxel sizes (i.e., higher matrix), thereby allowing fewer spins to co-exist per voxel. Another useful method is the use of a shorter TE. This allows a given spin less time to go out of phase from the time that they are tipped into the transverse plane until the signal is detected [6, 23]. Troubleshooting intravoxel dephasing can also be achieved using balanced steady-state free precession (b-SSFP), such as constructive interference in steady-state (CISS) and fast imaging employing steady-state acquisition with phase cycling (FIESTA-C). These imaging techniques employ gradients of polarity opposite to the slice selection, readout, and phase encoding directions (hence balanced), resulting in no net dephasing of the transverse magnetization for each TR [26]. In certain scenarios, intravoxel dephasing can be a critical imaging feature in diagnostic decision-making. An important example is this is in the evaluation of patency of the site of endoscopy third ventriculostomy (ETV), a procedure often performed as part of treatment for obstructive hydrocephalus. The presence of intravoxel dephasing through the floor of the third ventricle is a sign of a functionally patent ETV defect [27]. The role of FIESTA imaging in the context of intra-voxel dephasing is demonstrated in Fig. 10.

**Fig. 11:**
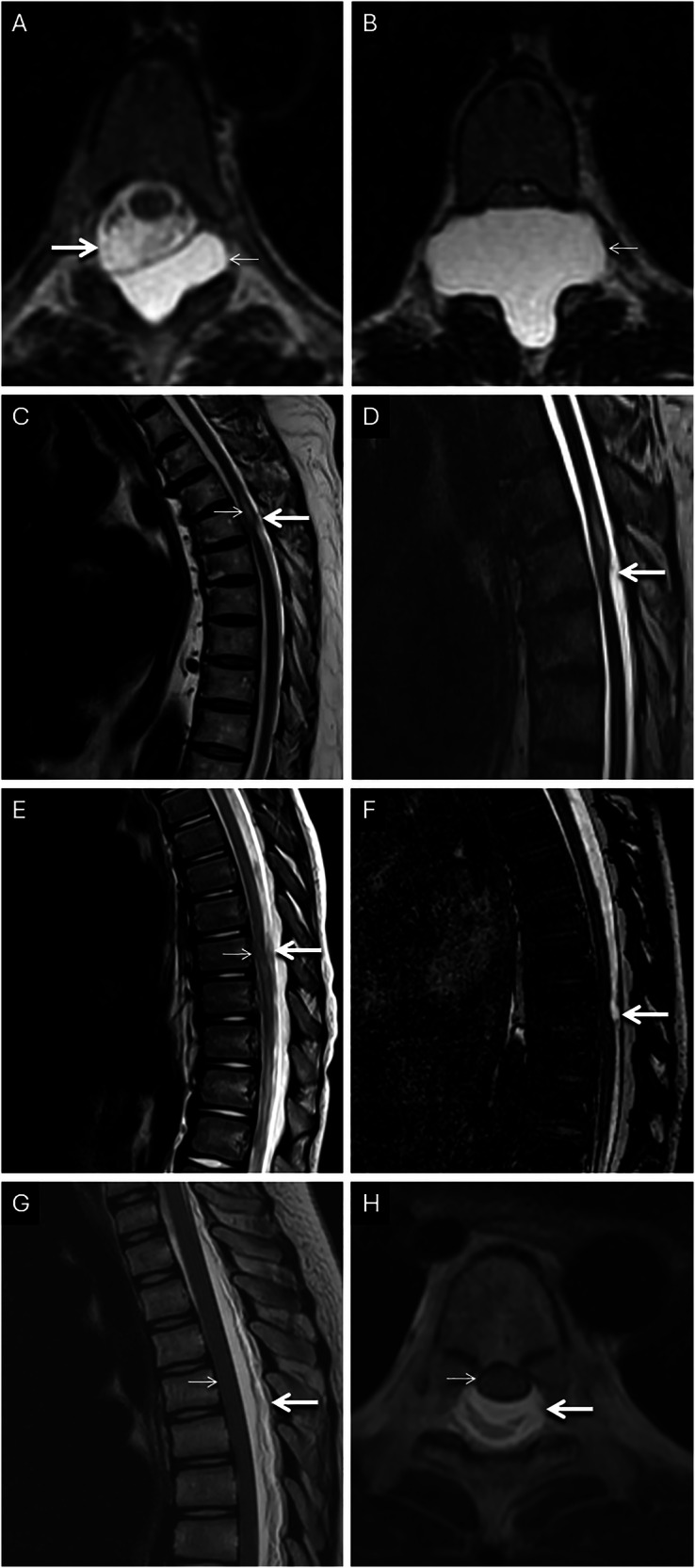
Utility of intravoxel dephasing. Patient 1: epidural arachnoid cyst in the thoracic spine (**A**, **B**) in a 15-year-old. Axial sequential craniocaudal T2W images (**A**, **B**) reveal a large lobulated arachnoid cyst (thin arrows) in the dorsal aspect of the thoracic spinal canal, devoid of dephasing artifacts (compared to the displaced thecal sac (arrow in **A**). Patient 2: thoracic dorsal arachnoid web (**C**, **D**) in a 38-year-old presenting with myelopathy. Sagittal T2W image (**C**) reveals a focal scalloping of the dorsal aspect of the cord in the upper thoracic region (i.e., scalpel sign) with proximal cord expansion and intrinsic T2 hyperintensity (thin arrow). Note the preserved intravoxel dephasing along the dorsal CSF column in this region (arrow). Sagittal FIESTA (**D**) obtained through the region of interest rectifies the CSF flow artifacts (arrow) and confirms the absence of a focal mass lesion in this location. An arachnoid web was identified during surgery (not shown). Patient 3: ventral cord herniation in a 3-year-old on the background of a previously repaired lumbosacral meningomyeolocele (**E**, **F**). Sagittal T2W image (**E**) reveals focal ventral kinking of the thoracic cord at the T8 level (thin arrow). Note the presence of intravoxel dephasing in the dorsal CSF column (arrow). Sagittal FIESTA (**F**) obtained through the region of interest confirms the absence of a focal lesion (in view of the rectification of the intravoxel dephasing; arrow) and the diagnosis of ventral cord herniation. Patient 4: 7-year-old presenting with a longitudinally extensive epidural collection following a lumbar puncture (**G**, **H**). Sagittal (**G**) and axial (**H**) T2W images reveal a large posterior epidural fluid collection in the thoracic spinal canal (arrows). Note the collection demonstrates hyperintense signals, however, it is completely devoid of intravoxel dephasing artifacts (as opposed to the appearances of normal, uninhibited flow of CSF) and exerts mass effect resulting in ventral displacement of the cord (thin arrows) and effacement of the thecal sac

The presence or absence of intravoxel dephasing in the spine can also be used as a surrogate for differentiating intraspinal cystic lesions such as arachnoid cysts, ventral cord herniations, and arachnoid webs. These conditions can cause anterior displacement of the spinal cord and are commonly encountered in the thoracic region. Arachnoid cysts have relatively stationary internal fluid and are devoid of artifacts relative to the surrounding mobile spins in flowing CSF. On the contrary, CSF flow around an anteriorly displaced spinal cord remains unimpeded in the setting of a thoracic arachnoid web or ventral cord herniation. Hence, intravoxel dephasing-related signal loss is seen within the widened subarachnoid space, dorsal to the displaced cord [28, 29]. The use of high-resolution, heavily T2-weighted sequences can further help differentiate between webs from cord herniations. Focal dorsal indentation of the cord (scalpel sign) and preservation of CSF ventral to the cord suggest the former, while effacement of the ventral CSF is noted in cases of cord herniation into a ventral dural defect [30]. In our experience and extrapolating from these concepts, spinal longitudinal epidural CSF collections can also be detected on T2-weighted images, by recognizing T2 hyperintense widening of the epidural space, devoid of turbulent flow artifacts, displacing the subarachnoid CSF column, demonstrating these artifacts. Examples of the diagnostic utility of intravoxel dephasing and FIESTA imaging are provided in Fig. 11.

## Conclusion

CSF flow artifacts are part of the day-to-day practice of neuroradiologists. They may degrade the image or help with image interpretation depending on the circumstances. Being familiar with the most common sequences and locations of these artifacts can prevent misdiagnosis and unnecessary further investigations.

## Data Availability

This manuscript is based on publicly available literature. No new datasets were analyzed or generated during the study. All data supporting the conclusions of this article are included within the article itself. Any additional material can be provided upon request to the corresponding author.

## Abbreviations

CSF: Cerebrospinal fluid
ESP: Entry slice phenomenon
ETV: Endoscopy third ventriculostomy
TE: Time to echo
TOF: Time-of-flight
TR: Repetition time
